# New and Specialized Methods of Image Compression

**DOI:** 10.3390/jimaging8020048

**Published:** 2022-02-16

**Authors:** Roman Starosolski

**Affiliations:** Department of Algorithmics and Software, Silesian University of Technology, 44-100 Gliwice, Poland; rstarosolski@polsl.pl

Due to the enormous amounts of images produced today, compression is crucial for consumer and professional (for instance, medical) picture archiving and communication systems. In response to practical needs, many algorithms and standards of image compression were developed. The most dynamic period in the development of image compression methods was at the turn of the century, when such algorithms were created as, for example, JPEG2000 with its plentitude of features targeted at diverse applications. Since then, several new image compression methods and algorithms were proposed as well as certain categories of images previously considered exotic have become popular and now demand efficient compression. Although currently the research community is more focused on the coding of video data, the image compression algorithms are constantly being improved and developed. Importantly, still image and video compression algorithms often exploit the same methods, and developments in one field can benefit the other.

The purpose of this Special Issue “New and Specialized Methods of Image Compression” was to provide a forum for new developments in the image compression domain. The focus was placed on promising image compression methods targeted at both typical photographic images and other image types that are increasingly used today. However, we also looked forward to contributions of research and overview papers concerned with methods targeted at related scientific fields that could be applied in image compression and with compression methods applicable in the related fields.

This Special Issue received several submissions, which underwent a rigorous peer-review process. After the review process, six articles (five research papers and one review article) were selected based on the ratings and comments. The published articles cover various applications of image compression methods characterized below.

In the work by Cheremkhin, Kurbatova, Evtikhiev, Krasnov, Rodin, and Starikov [1], a new method of digital hologram binarization was proposed. Hologram binarization is used in many domains including optical encryption, data compression, beam shaping, 3D displays, nanofabrication, materials characterization, and others. The proposed method was based on error diffusion, local thresholding, and block division. With respect to reconstruction quality, it outperformed standard binarization techniques both in numerical simulations and optical experiments.

The paper by Wang, Kosinka, and Telea [2] concerns medial descriptors that are used for image simplification, representation, manipulation, and compression. The Authors, based on the Compressing Dense Medial Descriptors scheme, introduced several improvements aimed at better compression ratios and reduced computational time and memory complexities; the main improvement was modeling medial descriptors with stable and accurate B-splines. In comparison to two other methods for various natural and synthetic images, depending on the image type, the proposed method allowed a very high compression ratio improvement at a cost of only a small quality loss. Additionally, the B-spline medial descriptor representation provides vector representation of the raster image that can be used for generating super-resolution images, and for compression that preserves salient features.

Prasetyo, Wicaksono Hari Prayuda, Hsia, and Guo [3] exploited deep learning to improve the subjective quality of decoded images compressed using the halftoning-based block truncation coding (H-BTC). They used convolutional neural networks and residual learning frameworks to reduce the amount of noise and suppress blocking artifacts in decoded H-BTC images. The effectiveness of the proposed method was assessed using both subjective and objective quality measures. As the method is applied as a post-processing step on a decoded image, it may potentially also be used for other than H-BTC lossy image compression methods.

Iqbal and Kwon [4] proposed to improve the JPEG algorithm by storing the location of end-of-block codes for empty blocks in a separate buffer and compressing the buffer with the lossless method, being either Huffman or arithmetic coding, which is used for the rest of the image data. As the result, at the same Peak Signal to Noise Ratio value, they achieved a higher compression ratio than the conventional JPEG encoder, whereas the level of improvement tended to be greater for Huffman coding and varied substantially between images. 

In [5], Ortis, Grisanti, Rundo, and Battiato addressed the problem of compression of stereoscopic images. After overviewing stereoscopic image compression, they investigated 16 variants of the Adaptive Stereoscopic Image Compression approach (resulting from using different optimization methods, different keypoint extraction techniques, and different compression ratios). Both objective and subjective compression quality measurements were employed in the evaluation of these variants; it was found that the method was able to obtain a high compression ratio of stereoscopic images while maintaining the visual quality. The Adaptive Stereoscopic Image Compression approach was also compared with other Multi-Picture Object compression methods.

Finally, Martínez-Rach, Migallón, López-Granado, Galiano, and Malumbres [6] present a comprehensive overview and comparison of the proposed successors of the High Efficiency Video Coding standard, namely the Joint Exploration Model (JEM) and Versatile Video Coding (VVC). These new algorithms, although designed mainly for video formats including Ultra High-Definition video and its different flavors (360°, AR/VR, etc.), are also effective for various kinds of still images. The comparisons were performed considering both the Rate/Distortion (R/D) performance and the complexity of the encoder—the two features that are the most important for practical applications. The obtained results showed that VVC is a better trade-off between R/D performance and the encoding complexity than JEM.
